# The Manufacturing of High Porosity Iron with an Ultra-Fine Microstructure via Free Pressureless Spark Plasma Sintering

**DOI:** 10.3390/ma9060495

**Published:** 2016-06-21

**Authors:** Guodong Cui, Xialu Wei, Eugene A. Olevsky, Randall M. German, Junying Chen

**Affiliations:** 1 School of Materials Science and Engineering, Southwest Jiaotong University, Chengdu 610031, China; chenjunying@swjtu.edu.cn; 2 College of Engineering, San Diego State University, 5500 Campanile Drive, San Diego, CA 92182, USA; xwei@mail.sdsu.edu (X.W.); randgerman@gmail.com (R.M.G.)

**Keywords:** porous iron, hollow Fe–N powder, free pressureless spark plasma sintering, compressive strength

## Abstract

High porosity (>40 vol %) iron specimens with micro- and nanoscale isotropic pores were fabricated by carrying out free pressureless spark plasma sintering (FPSPS) of submicron hollow Fe–N powders at 750 °C. Ultra-fine porous microstructures are obtained by imposing high heating rates during the preparation process. This specially designed approach not only avoids the extra procedures of adding and removing space holders during the formation of porous structures, but also triggers the continued phase transitions of the Fe–N system at relatively lower processing temperatures. The compressive strength and energy absorption characteristics of the FPSPS processed specimens are examined here to be correspondingly improved as a result of the refined microstructure.

## 1. Introduction

Porous metallic materials have attracted considerable attention because of their excellent structural and functional properties [1,2]. For porous materials with a similar level of porosity, smaller pores size can provide a larger specific surface and interfacial areas. Reducing pore size also helps to refine the microstructure and improve the mechanical properties [3]. In the past several decades, various porous metal materials have been developed and produced for the need of industrial applications, such as energy absorption [4,5], weight reduction, energy conservation [6], damping noise reduction [7,8], biomedical implants [9], and energy storage [10,11]. However, applications of porous metallic materials have been limited due to their low mechanical properties and complicated preparation process. In recent years, bulk iron-based porous materials have been considered the most promising porous materials due to their excellent mechanical properties, low cost, and extensive application backgrounds [2,12].

Most bulk porous iron-based materials are produced via casting or sintering processes [1,2]. Casting technologies include adding a blowing agent to the molten metal, freeze casting [13,14], and directional solidification in hydrogen, nitrogen, or argon atmosphere [15,16]. Sintering techniques are often used to fabricate isotropic porous metal materials. The porosity, pore size, and pore distribution can be easily controlled during the sintering process by adding pore-forming agents [1,2]. Commonly employed processes are mixing metal powders and space holders, pre-compaction in conventional powder press, removal of space holders (or pore-forming agents), and sintering [17,18]. These space holders or foaming agents include inorganic salt, organics, and titanium hydride (TiH_2_) [19,20,21]. As a matter of fact, environmentally harmful gases and residues might be released into the matrix during the removal of space holders, and the properties of the obtained final product could be negatively influenced [18]. To keep the impacts of space holder as few as possible, rapid sintering techniques have been used to fabricate metal foam materials from hollow metal particles and fibers, as they are able to achieve required densification level in short periods of time even without using space holders [22,23]. 

Spark plasma sintering (SPS), as an advanced sintering technology, is frequently used to consolidate various ceramic and metal materials at relatively lower temperatures [24,25]. Recently, this technique has been applied to produce porous materials through both free pressureless and conventional setups with the aid of dissolutions of inorganic salt (such as NaCl) [26,27]. Moreover, due to its rapid heating rate, this technique has also been widely applied in fabricating ultra-fine grained materials [28]. One recent study found that the iron nitride powders can be used to fabricate porous iron alloys with ultra-fine grains by conventional SPS, and that the continued Fe–N phase transition process has an obvious effect on grain refinement and pore formation during the sintering process [29]. This study also confirmed that rapid sintering technology is able to fabricate ultra-fine porous metal pellets using ultra-fine porous metal particles as raw materials. 

In this study, submicron-sized hollow Fe–N particles were used to fabricate ultra-fine porous iron specimens with high porosity but good mechanical properties via free pressureless spark plasma sintering (FPSPS) at a maximum sintering temperature of 750 °C. Since the hollow structured Fe–N powder is non-toxic, non-flammable, non-polluting, and chemically stable, the use of this powder as a pore-forming agent can bypass the procedure of adding and removing inorganic or organic space holders. The microstructure, phase composition, compressive properties, and energy absorption capability of the obtained products were evaluated and compared to previous reported data. The FPSPS manufacturing of ultra-fine porous iron is here shown to be simple, manageable, and environmentally friendly.

## 2. Results and Discussion

**Figure 1:**
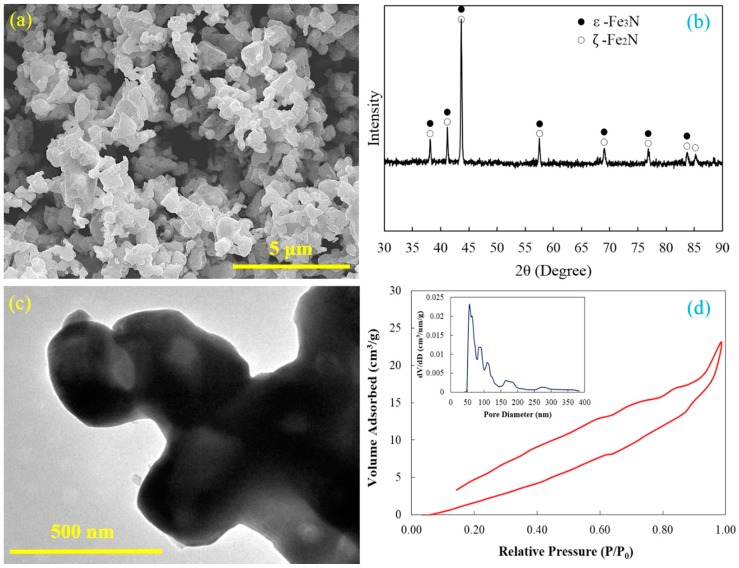
Hollow structure of Fe–N particles: (**a**) SEM image; (**b**) XRD pattern; (**c**) TEM image; (**d**) Adsorption–desorption isotherm and pore size distribution (inset) of Fe–N powder.

The synthesized Fe–N powders consist of uniformly submicron iron nitride particles and these particles are extremely agglomerated (Figure 1a). A few pores on the surface of Fe–N powders can be identified through careful examination. The *ε*-Fe_3_N and *ζ*-Fe_2_N are the main phase compositions of the Fe–N powder based on the X-ray diffraction pattern (Figure 1b). There are no peaks of iron oxide and iron presenting on the X-ray diffraction pattern, which indicates that all iron oxide powders have been completely reduced and nitrided by ammonia. The TEM investigation gives more details of morphological and structural features of the Fe–N powder. A typical TEM bright field image of agglomerated Fe–N powders is shown in Figure 1c. It can be seen that the Fe–N powder has an irregular geometrical shape and particle size ranging from 300 to 500 nm. Since there are brighter areas in the Fe–N particle, the Fe–N powder is shown to have a porous or hollow structure, as black areas usually indicate a fully dense structure in a TEM image. This porous structure was most likely formed during reduction and nitrodation reactions. A thin layer of nitrides was first generated on the powder surface, and the ammonia kept reacting with the internal substance by penetrating into the powder. Large volumes of gas were released during the reduction process, and these gases were not able to escape to the powder surface within a short period of time. Therefore, residual gas bubbles were trapped in the powder and formed the hollow porous structure. 

Figure 1d shows the nitrogen adsorption–desorption isotherm and a Barrett–Joyner–Halenda (BJH) pore size distribution of the Fe–N powders. The isotherm shows significant hysteresis, which also indicates the particular characteristics of the fine structure and strong adsorption of the powder. The strong adsorption observed at *P*/*P*_0_ close to 1.0 is a result of the accessible large pores in the Fe–N particles. The average pore size in the Fe–N powder was measured to be around 89.8 nm by the BJH method, which is in a good agreement with the above-mentioned TEM results. The maximum BET surface area and the maximum pore volume were 1.598 m^2^/g and 0.036 m^3^/g, respectively. All of the above-mentioned results support the fact that the Fe–N powder has a hollow porous structure and large specific surface area.

Table 1 summarizes the pre-compaction pressure, density, and porosity of green compacts as well as those of sintered specimens. Most pores were retained in the sintered specimens under the FPSPS conditions, and the porosity of the sintered specimens increased with decreasing pre-compaction pressure. After being sintered, approximately 10%–15% of the pores were eliminated with the volume shrinkage. The volume shrinkage mainly came from the reduction of inter-particle pores as a result of inter-particle neck formation and growth during FPSPS.

**Figure 2:**
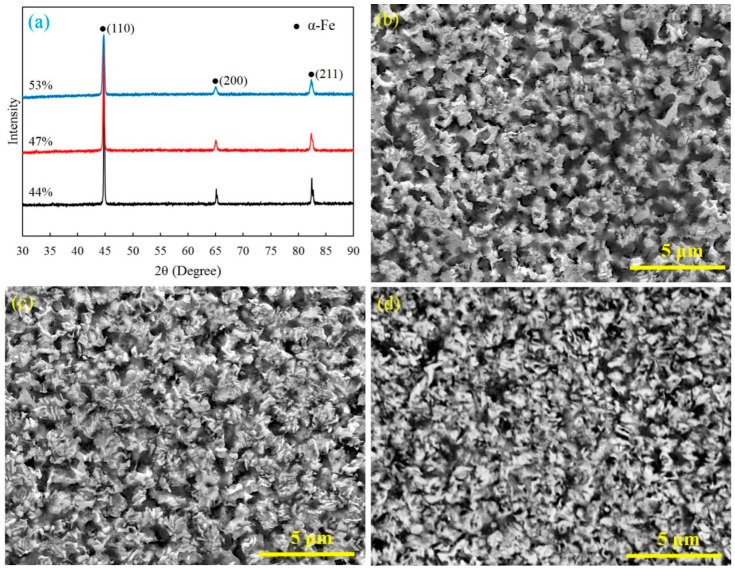
XRD pattern (**a**) and SEM micrographs of porous iron with different porosity: (**b**) 44%; (**c**) 47%; and (**d**) 53%.

Figure 2 shows the XRD patterns and SEM micrographs of the polished cross section of the sintered specimens. The main composition of the sintered specimen is *α*-Fe (Figure 2a). As shown in Figure 2b–d, the specimens sintered by FPSPS under different pressures have showed completely different microstructures and pore characteristics. A large number of isotropic pores on a micro- and nanoscale were formed and evenly distributed in the matrix materials, which effectively prevented grain growth and contributed to the finer framework structure.

As one can see, the inter-particle necks were easy to form and grow during the FPSPS process. The increase of pre-compaction pressure contributed to the formation of close-pore structures in sintered specimens (Figure 2b,c). Further, open-pore structures with micro-/nano-pores seemed to be easily observed in the specimens sintered from relatively lower density green compacts (Figure 2d). According to Figure 1b and Figure 2a, and the Fe–N phase transformation process [29], the Fe_2_N or Fe_3_N can gradually transform into Fe_4_N, Fe(N), and Fe as the sintering temperature increases to 750 °C. This transform process also indicates that the nitrogen gas can be produced continually during Fe–N phase transformation. This gas can help to facilitate the formation of pores and prevent grain growing if they are not released in time. Therefore, the porosities in sintered specimens mainly come from inner-particle and the Fe–N phase transformation process, while few come from the inter-particle. In addition, the rapid heating rate, the relatively lower sintering temperature, and the short holding time also contributed to the slow grain growth and facilitated the formation of the ultra-fine porous structure.

**Figure 3:**
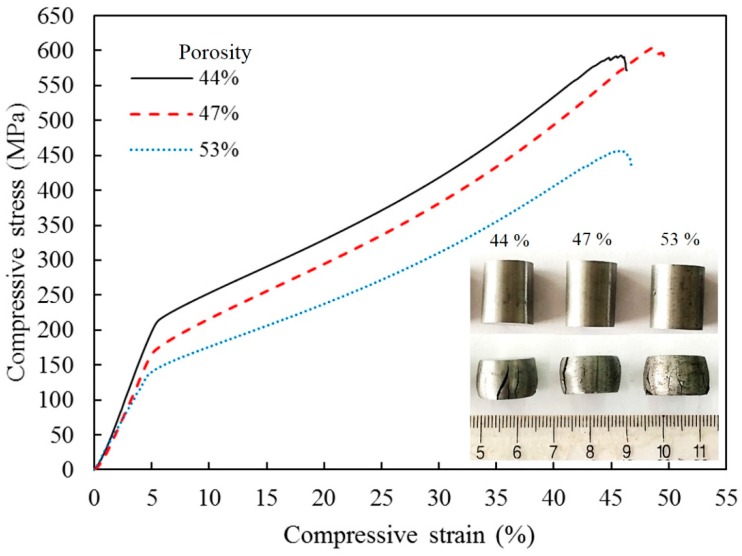
Room temperature uniaxial compressive stress–strain curves of porous iron prepared by pressureless SPS.

The mechanical properties of the ultra-fine microstructure porous iron were examined by uniaxial compressive tests at room temperature. The obtained compressive stress–strain curves are illustrated in Figure 3. These curves have the same evolution tendency and exhibit the typical behavior of ductile porous metal materials [17,18]. The difference is that these curves have not distinguished collapse plateau stage and are only characterized by two regions: In the first linear portion, the compressive stress increases rapidly with increasing strain until the yield point appears at a strain of about 4%. After yield, the compressive stress–strain curves of the porous sintered iron show a gentle ramping up stage, where the stress increases slowly in response to the increase in strain, which indicates a long-term limited deformation strengthening process [30].

The compressive properties and energy absorption properties of sintered specimens are shown in Table 2. It is apparent that either increasing relative density or decreasing porosity corresponds to an increase in Young’s modulus and yield strength of the sintered porous iron (Table 2). Young’s modulus was measured and calculated from reloading curves after unloading prior to visible plastic deformation. The compressive yield strength was measured as the intercept of tangents taken from the adjacent pre- and post-yield point of the stress–strain curve [17]. The compressive strength is strongly dependent upon the microstructure of the sintered specimens, and the ultra-fine microstructure improves the resistance capability of the porous iron with the bending and the buckling of the “struts”. In addition, the Young’s modulus of the sintered specimens increased from 3.14 GPa to 4.29 GPa with an increasing density from 3.69 g/cm^3^ to 4.40 g/cm^3^.

Room temperature compressive properties can be expressed, based on the Gibson–Ashby models utilizing the foam Young’s modulus $E_{f}$ and foam compressive yield strength $\sigma_{f}$, as Equations (1) and (2) [17].

(1) $$E_{f}=C_{E}E_{S}\left(\frac{\rho^{*}}{\rho_{S}}\right)m=C_{E}E_{S}{\left(1-p\right)}^{m}\;$$

(2) $$\sigma_{f}=C_{\sigma }\sigma_{s}{\left(\frac{\rho^{*}}{\rho_{s}}\right)}^{k}=C_{\sigma }\sigma_{s}{\left(1-p\right)}^{k}$$

where $\sigma_{s}$ and $E_{s}$ are the compressive yield strength and Young’s modulus of the bulk material, $\rho^{*}/\rho_{s}$ is the relative density of the foam, *p* is the porosity, *C* are the scaling factors, and *m* and *k* are the constants.

By fitting Equations (1) and (2) with the experimental data (Table 2), the constants in Equations (1) and (2) were optimized to represent the compressive Young’s modulus ($E_{f}$) and yield strength ($\sigma_{f}$) of the sintered specimens as a function of the relative density ($\rho^{*}/\rho_{s}$) to produce Equations (3) and (4).

(3) $$E_{f}=13.5{\left(\frac{\rho^{*}}{\rho_{s}}\right)}^{2}=13.5{\left(1-p\right)}^{2}$$

(4) $$\sigma_{f}=1250{\left(\frac{\rho^{*}}{\rho_{s}}\right)}^{3}=1250{\left(1-p\right)}^{3}$$

In Equation (3), the computed results are in a good agreement with the Gibson–Ashby models using a solid modulus $E_{s}$ of 200 GPa for iron or steel, for which the value of $C_{E}\approx 0.07$ is found, and the scaling factor ($C_{E}$) is lower than the magnitude of the reported values of the scaling factors of the Fe-based foams (0.1–0.3) [17]. This is an indication of a comparatively lower resistance to elastic deflection. From Equation (4), the fitting of the yield strength was not in good agreement with the Gibson–Ashby models. The resulting $C_{\sigma }\sigma_{s}=1250$ MPa, which fitted the experimental data, was much larger than the values of other Fe-based foams ($C_{\sigma }\sigma_{s}<345$ MPa) [17]. This indicates that the strength of the matrix material was greatly improved by refining the microstructure. Such an enhancement is directly related to the grain size, which is smaller in the case of the FPSPS-sintered Fe-based porous materials.

On the contrary, the large numbers of pores uniformly distributed in the iron matrix effectively prevented grain growth and contributed to the formation of a finer framework structure (Figure 2). Thus, the yield strength of the framework was improved remarkably by reducing the grain size. The energy absorption capacity per unit mass (W) and the energy absorption efficiency (η) were calculated from the compressive stress–strain curves (Figure 3) as follows [5]:

(5) $$W=\frac{\int_{0}^{\varepsilon_{m}}\sigma d\varepsilon }{\rho^{*}}$$

(6) $$\eta =\frac{\int_{0}^{\varepsilon_{m}}\sigma d\varepsilon }{\sigma_{m}\varepsilon_{m}}$$

where $\rho^{*}$ is the density of the porous iron, $\varepsilon_{m}$ is the given strain, $\sigma_{m}$ is the corresponding compressive stress, σ is the compressive stress as a function of strain ε, and *η* is the efficiency of the absorbed energy. The absorbed energy per unit mass and the efficiency of energy absorption of the sintered specimens during dynamic compression are shown in Table 2.

The energy absorption of the porous iron is higher than that of other sintered iron foams with isotropic pores (<30 kJ/kg) [30,31], whereas the energy absorption efficiency of the porous iron is close to 60%. This is mainly because of the higher yield strength and a wider strain range in the long gently stress region (Figure 3). In the dynamic compression of the sintered specimens with 44%, 47%, and 53% porosity at room temperature, the absorbed energy reaches 37.2, 39.08, and 32.57 kJ/kg, respectively.

These energy absorption characteristics of sintered specimens are caused by the two different deformation specifics originating from micro- and nanoscale isotropic pores and matrix metals. In general, for porous metals with isotropic pores, high absorbed energy and high energy absorption efficiency cannot be attained at the same time [30]. Generally, pores are considered defects in solid materials; however, a uniform distribution large number of micro- and nanoscale isotropic pores in the matrix can also have a strengthening effect on the matrix materials by preventing dislocation movement and inhibiting grain growth. These effects are very similar to dispersion strengthening or second-phase strengthening [32]. The energy absorption characteristics can be simultaneously improved along with the matrix strengthening.

## 3. Materials and Methods

**Figure 4:**
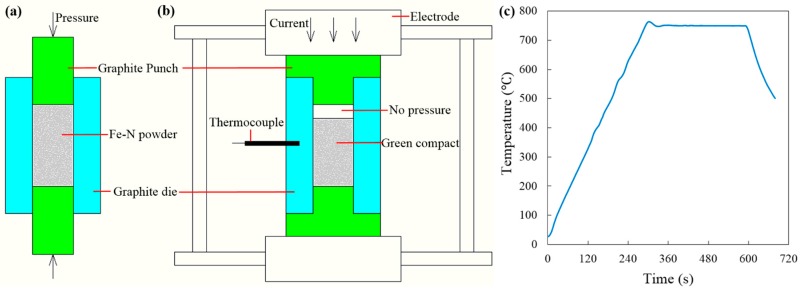
Schematics of the preparation process of porous iron. (**a**) Pre-compaction; (**b**) pressureless SPS process; (**c**) temperature profile used in the pressureless SPS process.

The Fe–N powder utilized in the present study was synthesized using ammonia reduction and nitridation of commercial iron oxide powders (99%, 300 nm, Chengdu Jingke Materials Ltd., Chengdu, China) at 600 °C for 3 h. The obtained Fe–N powder has an average particle size around 300–500 nm. Weighted Fe–N powders were poured into a 15.3-mm graphite die (I-85 graphite, Electrodes Inc., Santa Fe Spring, CA, USA), whose inner wall had been previously lined with 0.15-mm-thick graphitized paper. Two 15-mm cylindrical graphite punches were used to pre-compact the loaded powder at room temperature within the 15.3-mm die (see Figure 4a). In order to obtain green compacts with different initial densities, the Fe–N powders were pre-compacted under different axial pressures of 20 MPa, 40 MPa, and 60 MPa. After that, these cylindrical graphite punches were removed from the die, and two T-shape graphite punches were placed back to form the free pressureless SPS setup (see Figure 4b). All free pressureless SPS experiments were conducted in a vacuum using a Dr. Sinter SPSS-515 furnace (Fuji Electronic Industrial Co., Ltd., Kawasaki, Japan) [25].

The heating profile is illustrated in Figure 1c: The specimen was first heated up from room temperature to peak temperature at a heating rate of 150°/min and then followed by a 5-min isothermal holding stage in the vacuum (<1 Pa). A 3-kN minimum contact pressure between the die and the T-shape punches was maintained to ensure that the pulsed DC current could go through the tooling components and heat them up rapidly through the Joule heating effect [33]. The maximum processing temperature was selected as 750 °C, as ultra-fine porous structure could be obtained at this temperature according to the Fe–N phase transformation diagram in [29]. The real-time temperature during the SPS process was measured by a K-type thermocouple inserted into a 3-mm depth hole in the middle point of the lateral surface of the graphite die (Figure 4b).

The initial densities of the green compacts were calculated by means of a geometrical method, and the densities of the sintered specimens were measured by means of a water immersion method. The specific surface area (SSA) and pore size distribution of the raw Fe–N powders were determined by nitrogen adsorption–desorption at 77 K using Barrett–Joyner–Halenda (BJH) methods (Quadrasorb. S.I., Quantachrome Instruments, Boynton Beach, FL, USA) after degassing samples at 300 °C for 3 h. The microstructures of Fe–N powders were observed using transmission electron microscopy (TEM, JEM-2100F, JEOL Ltd., Tokyo, Japan) with an accelerating voltage of 200 kV. The microstructures of sintered specimens were observed using scanning electron microscopy (SEM, Quanta 450, FEI Corp., Hillsboro, OR, USA) after etching their cross-sectional areas with 5 vol % Nital. The phase composition of powder and sintered specimens were examined by X-ray diffraction (XRD, X’ pert pro, PANalytical B.V., Almelo, The Netherlands) with Cu K-alpha radiation. The Bragg angles were adjusted in the range of 30°–90° for the samples with a scanning rate of 5°/min. The compressive properties of sintered specimens were tested with a uniaxial compression test using a mechanical properties testing system (WDW-200, Changchun Kexin Test Instrument Co., Ltd., Chuangchun, China) with a loading rate of 5 mm/min. 

## 4. Conclusions

In summary, ultra-fine microstructure porous irons with high porosity (>40%) were successfully fabricated by free pressureless SPS at 750 °C using submicron hollow structured Fe–N particles as raw materials. The entire process was environmentally friendly by eliminating the procedures of extra adding and removing space holds. After rapid sintering, a large number of micro- and nano-scaled isotropic pores were formed and evenly distributed in the matrix materials. The continuous Fe–N phase transformation contributed to the formation of the ultra-fine porous structure. The high porosity in the sintered specimens mainly came from the pores in particles, and between particles, and produced during phase transitions in the Fe–N system. These micro- and nano-sized pores and phase transformations in the Fe–N system effectively inhibited grain growth at lower sintering temperatures and markedly refined the microstructure of the matrix materials. The compression stress–strain curves showed a high yield strength and wide strain range with a smooth plateau. Consequently, the energy absorption capability and efficiency were largely improved compared to other metallic foams with isotropic pores.

## Figures and Tables

**Table 1:** The pre-compacted pressure, density, and porosity of green compacts and sintered specimens.

Pre-Compacted Pressure, MPa	Green Compact Density, G/Cm^3^	Porosity of Green Compacts, %	Sintered Specimen Density, G/Cm^3^	Porosity of Sintered Specimens, %
20	2.5	64	3.69	53
40	3.0	57	4.17	47
60	3.2	54	4.40	44

**Table 2:** The compressive properties and energy absorption properties of porous iron sintered by free pressureless SPS.

Porosity %	Young’s Modulus, GPa	Yield Strength, MPa	Compressive Strength, MPa	Maximum Strain, %	W kJ/kg	*η* %
44	4.29	223.1	593.0	45.9	37.20	60.0
47	3.83	178.8	602.0	48.7	39.08	55.6
53	3.14	134.7	456.9	45.8	32.57	57.6
